# Supplementary material for: Tackling syndemics by integrating infectious and noncommunicable diseases in health systems of low- and middle-income countries: A narrative systematic review
Source: PLOS Glob Public Health. 2024 May 16;4(5):e0003114. doi: 10.1371/journal.pgph.0003114 (PMC11098501; doi:10.1371/journal.pgph.0003114)
Supplement: S1 Table — (DOCX) [file pgph.0003114.s001.docx]

# **S1 Table.** Ranking of evidence of included studies.

| **Ranking of evidence: Low: <40% Moderate: 40-80%, High: >80%** | |
| --- | --- |
|  |  |
| **TB and Diabetes Studies** |  |
| *Analytical Cross-Sectional Study* |  |
| **Citation** |  |
| Anand et al. 2018. | Moderate |
| Asante-Poku et al. 2019. | Moderate |
| Berkowitz et al. 2018. | High |
| Chamie et al. 2012. | High |
| Deepak et al. 2018. | High |
| Huangfu et al. 2019. | High |
| Jerene et al. 2017 | High |
| Li et al. 2012 | Moderate |
| Munseri et al. 2019 | High |
| Nair S et al. 2013. | High |
| Restrepo et al. 2011 | Moderate |
| Sarker et al. 2016. | Moderate |
| Segafredo, et al. 2019. | High |
| Sinha et al. 2018 | Moderate |
| Ugoeze et al. 2020 | Moderate |
| Zhang et al. 2015 | Moderate |
| *Cohort Study* |  |
| **Citation** |  |
| Faurholt-Jepsen et al. 2012. | High |
| Gnanasan yet al. 2011 | Low |
| Khanna et al. 2013. | Moderate |
| Kornfeld et al. 2016. | High |
| Mishra et al. 2020. | Moderate |
| Mukhtar et al. 2018. | High |
| Mukhtar et al. 2017. | High |
| Rekha et al. 2007. | High |
| *Diagnostic Test Accuracy* |  |
| **Citation** |  |
| Habib et al. 2020 | Low |
| *Prevalence Study* |  |
| **Citation** |  |
| Achanta et al. 2013 | High |
| Basir et al.2019 | Moderate |
| Chachra et al. 2014 | Moderate |
| Contreras et al. 2017 | Moderate |
| Ekeke et al. 2020. | High |
| Kumpatla et al. 2013. | Moderate |
| Naik et al. 2013. | High |
| Ncube et al. 2019. | High |
| Nimkar et al. 2020 | Moderate |
| Prakash et al. 2013. | High |
| Sarvamangala et al. 2014. | Low |
| Shayo et al. 2019 | High |
| *Qualitative Research* |  |
| **Citation** |  |
| Salifu et al. 2020. | Moderate |
| *Text and Opinion Study* |  |
| **Citation** |  |
| Brey et al. 2020 | High |
|  |  |
| **COVID and Diabetes. Obesity and CVD studies** |  |
| *Analytical Cross-Sectional Study* |  |
| **Citation** |  |
| Anjana et al. 2020 | Moderate |
| Catic et al 2020 | Moderate |
| Di Tommaso et al. 2020 | Moderate |
| Ding L et al. 2020. | Moderate |
| Ghosh et al. 2020. | Moderate |
| Gona et al. 2020. | Moderate |
| Joshi et al. 2020 | Moderate |
| Li et al. 2020 | Moderate |
| Olickal et al. 2020 | Moderate |
| Queiroz et al. 2020 | Moderate |
| Zhao et al. 2020 | Moderate |
| *Quasi-Experimental Study* |  |
| **Citation** |  |
| Nan et al. 2020 | Moderate |
| *Case Reports* |  |
| **Citation** |  |
| Concepción et al. 2020 | Moderate |
| *Text and Opinion Study* |  |
| **Citation** |  |
| Brey et al. 2020 | High |
| Cheng et al. 2020 | High |
| Co et al. 2020 | High |
| Krisiunas et al. 2020 | High |
| Liu et al. 2020 | High |
