# Supplementary material for: Tackling syndemics by integrating infectious and noncommunicable diseases in health systems of low- and middle-income countries: A narrative systematic review
Source: PLOS Glob Public Health. 2024 May 16;4(5):e0003114. doi: 10.1371/journal.pgph.0003114 (PMC11098501; doi:10.1371/journal.pgph.0003114)
Supplement: Attachment — Submitted filename: Review response basic text PLOS GH Integration Review.docx [file pgph.0003114.s002.docx]

Additional Editor Comments: Inclusion of HIV/NCD integration data to enhance the narrative. Please provide justifications for the infectious diseases selected for the study and provide a clearer distinction between the management of endemic and epidemic diseases. The reviewers have suggested to refine conclusions to align with the evidence presented. Also, please discuss how the exclusion of non-English language studies might impact the results.
We have clarified the rationale for excluding studies that examine integration of integrated health systems for NCDs and HIV. We believe there is substantial literature on integration for this purpose, including an entire special edition of the Journal of AIDS and a multi-country portfolio of NIH research projects ([e.g.](https://www.hlbsimple.org/) NHLBI-SIMPLE). Therefore we focused our inquiry on NCD co-morbidities that have received less attention. Nonetheless, given the importance of NCD-HIV comorbidities, we have sought to bring in some key points linking to findings from the NCD-HIV integration literature. These include, for example, the continuing need for more robust evaluation evidence in the HIV literature to better assess what effective and cost-effective integration looks like, and investment in HIV integration that goes beyond service delivery, which is key to more sustainable integration efforts.

We have indicated the exclusion of non-English language studies as a limitation and acknowledge that there may be studies published in other languages thereby missed.

4. Is the manuscript presented in an intelligible fashion and written in standard English?

Reviewer #1: No

We have further proof-read and edited the manuscript.

Reviewer #2: Yes

5. Review Comments to the Author

Reviewer #1: 

Introduction
The rationale of the review needs to be presented in detail.
Why is it important to integrate?
What are the gaps in existing evidence?

The text in the methods section now justifies the literature, as follows: ‘We did not search for integration evidence that included HIV and AIDS, as there is plentiful literature.9–14 Although we did not expect to find many studies, we searched for evidence of integration between NCDs and neglected tropical diseases (NTDs) due to NTD endemicity in many LMICs and the current policy attention to NTD eradication and/or containment goals. If found, we believed that such examples of integration could inform those policy discussions in countries with high NCD burdens. Recognizing the recent attention to interactions between epidemic COVID-19 and NCDs, we also sought lessons from integrating care for endemic IDs with comorbid NCDs, but no literature was identified concerning this dyad. The evidence we found was concentrated on the dyads of tuberculosis and diabetes and comorbid COVID-19 with multiple NCDs. We concentrated on relevant interventions across the WHO health system building blocks for adults in LMICs living with ID and NCDs.’

In paragraph one we explain the importance of addressing syndemic diseases and the benefits this can have for patient care: ‘Co-occurrence of infectious diseases (IDs) and non-communicable diseases (NCD) in patients is more common than in the past as demographic and epidemiological transitions affect most countries. More patients with comorbidities call upon health systems to offer a wider service package, gather additional data, and train health professionals to detect and manage complex disease combinations.1 While diseases differ in their pathogenesis, some IDs and NCDs share patient or risk factors and hence can be managed in similar ways.2 In addition, the underlying socio-economic determinants and health disparities may be similar despite differing causes. COVID-19 is becoming the iconic example of an ID that especially affects people living with chronic disease. A nascent literature on syndemic disease—defined as clusters of two or more socially-driven diseases, the interaction of which yields worse health outcomes than those diseases individually—highlights the need to understand comorbid disease through a public policy lens.3–6’

In paragraph two we explain that health managers are seeking solutions to be able to manage the burden of multi-morbidity and to adapt existing models of chronic care: ‘LMIC health system managers are seeking a paradigm shift to design and adopt chronic care models, integrate essential medicine supply chains, and integrate health information systems, while leveraging the existing infrastructure and health workforce that has little training in chronic disease.’

Methods
In the analysis section describe how did you prepare the Tree map of Health System Outcomes identified in the literature (as per the WHO building blocks).

Thank you for highlighting this omission. We have added explanatory text in a short paragraph in the methods section: ‘To analyze and present the results of this review, we prepared tree maps of Health System Outcomes identified in the literature as per the WHO building blocks. These maps were created using Excel and show the number of articles represented in each health system outcome.’

Provide a single search strategy for all domains.

We now include a single search strategy as recommended.

Results
Three PRISMA flow diagrams in a single review are confusing and unnecessary increasing the number of figures and tables. Make a single search, and in PRISMA provide the final how many articles were included:
Total studies included (N=) and in these articles
 A. TB and diabetes integration (n=)
 B. COVID-19 and Diabetes, Obesity, and CVD Integration (n=)
 C. COVID-19, Tuberculosis, and Diabetes Integration (n=)
Instead of three Tables on study characteristics, provide a single table with all the included studies. Combine the following tables into a single table. Create one more column on the domain address (A, B, C, AB, BC, CD, ABC).
Table 2: Baseline characteristics of included studies for tuberculosis and diabetes integration
Table 5: Summary of COVID-19 and Diabetes, Obesity and CVD integration articles by study characteristics
Table 7: Summary of COVID-19, Tuberculosis, and Diabetes integration articles by study characteristics.
Combine the three Maps of the included studies (figures 3, 6 and 9).
Present the figures 4, 7 and 10 (Tree Maps) separately (as it is provided).

We have developed a single tree diagram as recommended (Figure 1) and inserted text above, and also a single table of study characteristics (Table 2).

Discuss how the integration is cost-effective.
We have added to the TB-Diabetes section as follows: ‘Cost-effectiveness is a necessary aspect of health system interventions. There has been a strong presumption that integrated interventions would be cost-effective, relative to separate disease management. Yet, the economic evidence of greater cost-effectiveness is sparse and exclusively related to integrated care for HIV-positive people. We explored the key outcomes in that literature for indications of key research gaps.’

We have added to the Discussion section as follows: ‘Greater cost-effectiveness has also been posited to be a benefit of integration, but the evidence is insufficient to draw conclusions.’

Write a paragraph on research gaps and implications of the findings for policy and practices.

In the discussion we set out the research gaps for the conditions we studied in our review as follows:

Paragraph 1: ‘The limited literature found by this review across a range of major infectious disease and noncommunicable disease (NCD) dyads suggests that, prior to the COVID-19 pandemic, health system ID-NCD integration for non-HIV conditions was substantially limited in low- and middle- income countries. Evidence on NCD - trachoma / onchocerciasis integration was wholly absent, and tuberculosis and diabetes integration efforts predominated. For tuberculosis and diabetes integration there were few studies in low-income countries (LICs) and limited literature in middle income countries (MICs) – only 56 across the search period.’

Paragraph 3:’ Nonetheless, a continuing and significant limitation of the identified examples of integration for these conditions was their scope. Examples focused almost exclusively on service delivery, suggesting this has been prioritized rather than integration across health system building blocks, such as integrated workforce capacities or systems, financing, or essential medicines provision. This applied to all ID-NCD dyads in the study. Regarding tuberculosis and diabetes, the scope was even narrower, as service delivery integration almost wholly concentrated upon screening. Few examples addressed treatment beyond initial screening, with care thus continuing to be separate for the conditions.’

Reviewer #2: 

1. The decision not to integrate and include data on HIV/NCD integration is a major omission, since that is the area with the most literature. As such, comparative/narrative synthesis across NCDs is missing the area of literature with the largest body of experience. This HIV literature is not even engaged with in the discussion.

We have clarified the rationale for excluding HIV studies, in the Methods section, as follows: ‘We believe there is substantial literature on integration for this purpose, including an entire special edition of the Journal of AIDS and a multi-country portfolio of NIH research projects ([e.g.](https://www.hlbsimple.org/) NHLBI-SIMPLE). Therefore we focused our inquiry on NCD co-morbidities that have received less attention.’

Nonetheless, given the importance of NCD-HIV comorbidities, we have sought to bring in some key points linking to findings from the NCD-HIV integration literature, and indicating common gaps with the conditions we assessed in the Discussion: ‘A service delivery focus is apparent also in the more plentiful examples of countries’ HIV-NCD efforts, yet these commonly extend far more extensively into care delivery, and often encompass preventive services also.’

In the Conclusions: ‘Implementation projects – including regarding wider health system building blocks (a gap also noted in the HIV literature), and robust outcome and process evaluation are needed for countries to assess and maximize the potential for integrated approaches to manage infectious and noncommunicable diseases.’

2. The choice of infectious disease entities chosen is not justified and is idiosyncratic. There is very little literature, at all, on onchocerca and trachoma so the review becomes by default a review of TB alone. There are many other high impact infectious diseases, such as malaria, that could have be included.

The text in the methods section now justifies the literature, as follows: ‘We did not search for integration evidence that included HIV and AIDS, as there is plentiful literature.^9–14^ Although we did not expect to find many studies, we searched for evidence of integration between NCDs and neglected tropical diseases (NTDs) due to NTD endemicity in many LMICs and strong policy attention upon eradication and/or containment goals. While recognizing malaria as a high impact condition, we opted not to prioritize this, given the review focus on health system building blocks whereas many malaria interventions are outside the health system. Recognizing the recent attention to interactions between epidemic COVID-19 and NCDs, we sought lessons learned from integrating endemic IDs with NCDs as a counterpart, but no literature was identified concerning this dyad. The evidence we found was concentrated on the dyads of tuberculosis and diabetes, and comorbid COVID-19 with multiple NCDs.’

3. I question the taxonomic decision to include management of endemic infectious diseases, eg tuberculosis, within the same framework as epidemic infectious diseases (COVID). These are completely different phenomena.

Thank you for this comment, we appreciate that it is important to clearly justify the selection as our condition dyads are perhaps not the most obvious choice – and that is in part why we selected them. So we have explained and justified our selection in the revised text (pls see answer 2 above.)

4. Along with point 3, while much of the literature related to TB integration do represent true integrations of service lines the COVID related literature is much more about pandemic response adaptations which are not specific to NCDs but occurred across all areas of all health systems globally. These pandemic response adaptations are a distinct category and do not convincingly inform the management of both NCDs and endemic infectious diseases within primary care or integrated care models.

We have justified the inclusion in the revised text as per our answer to 2 above. In the Discussion we have also modified the text to (more) cautiously suggest that the at least limited consideration of IDs within NCD service delivery during the pandemic may at least provide a foundation to build upon: ‘Many NCD service delivery programs introduced measures related to the infectious disease COVID-19 during the pandemic. This greatly increased the number of published examples of programs taking an integrated approach to managing one or more NCDs and an infectious disease. The scope of integration followed the same pattern as for tuberculosis or diabetes, with health service delivery integration predominant (42/48 studies) – influenced by the high policy priority upon maintaining essential NCD services. Measures focused on reducing COVID-19 exposure risk and maintaining routine care access for NCD patients. In this way the pandemic - in many cases for the first time - encouraged health service staff in at least a limited way to consider IDs and NCDs together, and in many cases this integrated consideration has persisted, for example, in the continued greater focus given to infection control within NCD service delivery. The pandemic also produced a substantial research output indicating that untreated NCDs represent vulnerabilities that result in poorer health outcomes, and recommended this be considered within future pandemic planning.127–129 We propose therefore that COVID-19 may have a legacy in terms of awareness and willingness to consider integrated approaches to address co-occurring health challenges.’

5. The conclusions on the reach and distribution of ID-NCD integration are over-reaching, given the limited search strategies used.

We have adjusted the Discussion and conclusions to clearly reflect the review focus on ID-NCD integration for selected condition dyads, and we have drawn parallels where the gaps in integration evidence between HIV and the literature on the infectious diseases of focus are highly similar and both require future focus.

6. The conclusion that ID-NCD integration surged after COVID-19 is also over-reaching, since TB-NCD integration and similar scenarios for other endemic infectious diseases is a completely different phenomenon from the pandemic response and these two cannot be categorized together.

We have adjusted the text as per the answer under 4 above to more cautiously suggest that the consideration of an ID, sometimes for the first time, by NCD service providers and planners, may at least represent a foundation upon which further integrated care / system developments might build.

7. The exclusion of studies in languages other than English is a major limitation, and it is not clearly justified given how simple text translation has become using multiple research tools. Although not clear from the methods, the flowcharts seem to show that the language exclusion was applied at the level of the primary search, so we are not even given information on how many identifiable non-English language studies may be excluded here from the analysis.

Prior to the review we were aware that examination of NCD integration is at the ‘cutting edge’ of NCD research, and so based on co-authors’ knowledge of the HIV literature, we judged that at least in this early phase, most peer-reviewed integration studies would be published in English language journals. Had our scope also prioritized grey literature, we would expect more non-English reports. Nonetheless we have included the exclusion of non-English language studies explicitly as a limitation, and acknowledge that there may be some studies thereby missed.
